# Obesity, estrogens and adipose tissue dysfunction – implications for pulmonary arterial hypertension

**DOI:** 10.1177/2045894020952023

**Published:** 2020-09-18

**Authors:** Kirsty M. Mair, Rosemary Gaw, Margaret R. MacLean

**Affiliations:** Strathclyde Institute of Pharmacy and Biomedical Sciences (SIPBS), University of Strathclyde, Glasgow, UK

**Keywords:** aromatase, metabolic syndrome, sex differences

## Abstract

Obesity is a prevalent global public health issue characterized by excess body fat. Adipose tissue is now recognized as an important endocrine organ releasing an abundance of bioactive adipokines including, but not limited to, leptin, adiponectin and resistin. Obesity is a common comorbidity amongst pulmonary arterial hypertension patients, with 30% to 40% reported as obese, independent of other comorbidities associated with pulmonary arterial hypertension (e.g. obstructive sleep apnoea). An ‘obesity paradox’ has been observed, where obesity has been associated with subclinical right ventricular dysfunction but paradoxically may confer a protective effect on right ventricular function once pulmonary hypertension develops. Obesity and pulmonary arterial hypertension share multiple pathophysiological mechanisms including inflammation, oxidative stress, elevated leptin (proinflammatory) and reduced adiponectin (anti-inflammatory). The female prevalence of pulmonary arterial hypertension has instigated the hypothesis that estrogens may play a causative role in its development. Adipose tissue, a major site for storage and metabolism of sex steroids, is the primary source of estrogens and circulating estrogens levels which are elevated in postmenopausal women and men with pulmonary arterial hypertension. This review discusses the functions of adipose tissue in both health and obesity and the links between obesity and pulmonary arterial hypertension. Shared pathophysiological mechanisms and the contribution of specific fat depots, metabolic and sex-dependent differences are discussed.

## Introduction

Obesity is a growing public health problem worldwide, and its rising prevalence will likely contribute to an increased burden from several diseases, notably, cardiovascular disease, diabetes and cancer. Comorbidities are common amongst pulmonary arterial hypertension (PAH) patients, and 30% to 40% of patients with PAH are reported as obese.^1–3^ Obesity has been linked to sleep-disordered breathing and hypoventilation which may contribute to hypertensive changes in the pulmonary circulation. Indeed, histological changes indicative of pulmonary arterial and venous hypertension are present in a number of obese individuals without PAH.^4^ Furthermore, adipose tissue is now recognized as an important endocrine organ, and the excess accumulation of body fat that occurs in obesity can result in chronic low-grade inflammation, insulin resistance and adipose tissue dysfunction that may contribute to the pathogenesis of PAH.

Adipose tissue dysfunction in obesity results in a marked change in the profile of bioactive mediators produced. The altered secretion of such mediators from adipose tissue can contribute both directly and indirectly to the development of obesity-related diseases.^5^ Of interest, adipose tissue synthesizes estrogen, and adipose tissue expansion in obesity is known to contribute to development of estrogen-sensitive breast cancer.^6^ Adipose tissue contains high levels of the estrogen-metabolizing enzyme cytochrome P450 1B1 (CYP1B1), and in obese mice, thoracic adipose tissue produces 16α-hydroxyestrone (16OHE1),^7^ a known mitogen in the pulmonary circulation.^8^ It is also thought that 16OHE1 can increase the penetrance of mutations in bone morphogenetic protein receptor 2 (BMPR2) both experimentally and clinically.^9^

Mechanisms associating obesity to PAH are not well understood; however, both conditions share several underlying pathological mechanisms. The metabolic changes that arise in obese individuals may contribute to the development of a pathophysiological environment that facilitates pulmonary vascular remodelling contributing to the development and/or exacerbation of PAH. This review will focus on adipose tissue dysfunction in obesity, adipose-derived estrogens, sex-dependent differences and the contribution of specific fat depots and their role in PAH development and disease pathobiology.

## Obesity and adipose tissue dysfunction

Obesity is characterized by an excessive amount of body fat and an increased body mass index (BMI) of 30 kg/m^2^ and above. The composition of adipose tissue dramatically changes in obesity, involving alterations in adipocyte size and number, immune cell content and extracellular matrix resulting in a predisposition to metabolic dysfunction.

**Fig. 1.**
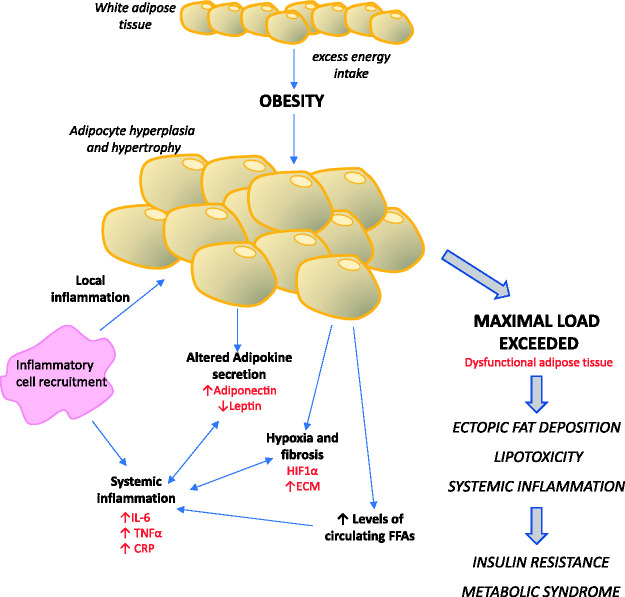
Adipose tissue dysfunction in obesity. Insufficient storage capacity of adipocytes in obesity results in systemic inflammation, increased lipolysis, hypoxia and altered adipokine secretion. These factors result in the development of insulin resistance and metabolic syndrome which further impairs adipose tissue function and contributes to obesity-related diseases. CRP: C-reactive protein; ECM: extracellular matrix; FFAs: free fatty acids; HIF1α: hypoxia-inducible factor-1; IL-6: interleukin-6; TNFα: tumour necrosis factor-α.

Chronic availability of excess nutrients leads to the expansion of fat depots, weight gain and obesity. Adipose tissue can expand by two main mechanisms: hypertrophy and hyperplasia. Hypertrophy involves increasing the size of individual adipocytes, whilst hyperplasia recruits new adipocytes from a reservoir of resident progenitor cells. The capacity of adipose tissue to expand is critical for accommodating changes in energy availability, but this capacity is not an unlimited process and varies between individuals.^10^ In obese humans, hypertrophy of adipocytes correlates with dyslipidemia, impaired glucose homeostasis and inflammation. Conversely, adipocyte size was found to be smaller in obese individuals without metabolic disease compared to those with metabolic complications.^11^ These observations suggest that large adipocytes are pathogenic, and/or the inability of adipocytes to further expand limits the capacity for excess lipid storage resulting in systemically elevated lipid levels. Therefore, a lack of hyperplasia, coupled with a high prevalence of hypertrophic adipocytes, results in a limited capacity of adipose tissue to expand and store fat. An individual’s capacity for adipose expansion is determined by both genetic and environmental factors, and once the adipose tissue expansion limit is reached, adipose tissue ceases to store energy efficiently.^12^ When the storage capacity of adipose tissue is exceeded, lipids can no longer be safely cleared from the systemic circulation, and the excess of circulating free fatty acids will be deposited in non-adipose organs including the liver, skeletal muscle, heart and the pancreas.^12,13^ This is known as lipotoxicity and accounts for many of the adverse effects of obesity, particularly changes in adipokine release and a low-grade inflammatory response that ultimately lead to metabolic dysfunction including reduced insulin sensitivity and glucose intolerance (Fig. 1).^12^

Healthy adipose tissue is highly vascularized, and each adipocyte is nourished by an extensive capillary network. However, the hypertrophic expansion of adipose tissue in obesity is often accompanied by inadequate angiogenesis leading to reduced capillary density and local hypoxia.^14^ Hypoxia is one of the first pathological changes to occur in adipose tissue during obesity and is thought to be a main driver of fibrosis via the activation of hypoxia-inducible factor 1 (HIF1α) and contributes to local inflammation and dyslipidemia (Fig. 1).^12,15,16^

Adipose tissue dysfunction in obesity results in a shift from an anti-inflammatory towards a proinflammatory profile. Obesity-associated inflammation starts in adipose tissue and liver with elevated macrophage infiltration and expression of proinflammatory cytokines. The inflammatory response triggered by obesity also results in an increase in the circulating levels of inflammatory cytokines such as interleukin (IL)-6 and tumour necrosis factor-α (TNFα), as well as increased acute phase proteins; C-reactive protein and serum amyloid A and causes systemic inflammation.^13^ Thus, overloaded, dysfunctional adipose tissue is associated with the activation of immune cells and inflammatory mediators both locally in adipose tissue and systemically resulting in a chronic, low-grade, inflammatory state (Fig. 1).

In addition to chronic inflammation, adipocyte overloading and lipotoxicity in obesity has a major impact on adipose tissue function, resulting in an adverse adipokine profile (Fig. 1). In particular, a reduction in adiponectin production is thought to be a major pathogenic factor in metabolic disease.^17,18^ Conversely, levels of the proinflammatory adipokine leptin are increased in obesity.^19,20^

The excess supply of energy substrates in obesity is also believed to lead to increased mitochondrial dysfunction and reactive oxygen species (ROS) signalling, resulting in cellular oxidative stress and an impact on the endocrine and metabolic function of fat cells.^21^ Obese individuals exhibit higher levels of oxidative stress in white adipose tissue, including elevated ROS levels and decreased antioxidant activity coupled with alterations in adipokines required for insulin sensitivity.^22^ Thus, the oxidizing environment in adipose tissue of obese individuals impacts fat cell function and energy balance.

Therefore, the excess adiposity that occurs in obesity is associated with adipose dysfunction which in turn contributes directly or indirectly to the development of obesity-related diseases. The accumulation of excessive visceral fat is accompanied by alterations at hormonal, inflammatory and endothelial level and as such can result in development of a variety of diseases including diabetes, liver disease and cancers. Obesity is also associated with the prevalence of most cardiovascular diseases, including systemic hypertension, coronary heart disease and PAH.

## PAH and obesity

Epidemiological evidence supports a link between obesity and PAH. Findings from the Registry to Evaluate Early and Long-Term PAH Disease Management (REVEAL), the largest pulmonary hypertension (PH) database in the United States, indicate that 32% of PAH patients are obese. Furthermore, a higher prevalence of overweight and obese individuals among those with idiopathic forms of PAH was observed.^2^ This association appears independent of conditions associated with the development of PAH (e.g. diastolic dysfunction, obstructive sleep apnea (OSA)), suggesting that obesity globally disrupts vascular homeostasis and predisposes individuals to the development of systemic and pulmonary vascular diseases. Similarly, in the Scottish Pulmonary Vascular Unit incident PAH population and a French multicentre population, 35.7% and 30% of patients were recorded as being obese, respectively.^1,3^ A greater portion of the obese PAH patients recorded were female.

Obesity has been associated with a significantly worse 6-minute walk time, functional class and haemodynamic parameters in PAH patients.^1–3^ Indeed, a link between body weight and the pulmonary vascular function appears to exist. A correlation between BMI and pulmonary artery systolic pressure has been identified in otherwise echocardiographically healthy individuals.^23,24^ In other studies, BMI significantly correlates with right atrial pressure and pulmonary artery pressure in incident, treatment naive PAH patients.^1^ However, some limitations of these studies must be acknowledged as only BMI was assessed, and they do not allow for differences in adipose tissue distribution or oedema.

The benefits of weight loss have also been demonstrated in obese PAH patients. Weight loss following bariatric surgery can result in an improvement in functional class and tolerance to exercise despite no changes to the PAH therapy administered. This weight loss also contributed to the inhibition of mechanisms underlying obese pathology resulting in reduction of insulin resistance, high-density lipoprotein (HDL), low-density lipoprotein and free fatty acids that correlate with haemodynamic improvements.^25^

### PAH and the obesity paradox

Obesity is a well-established independent risk factor for the development of cardiovascular disease and mortality. Nevertheless, substantial data have demonstrated an ‘obesity paradox’, where obese patients generally have a better short- and long-term prognosis than their leaner counterparts with the same cardiovascular diseases.^26^ In keeping with this observation, obesity has been associated with subclinical right ventricular (RV) dysfunction, but paradoxically, it may confer a protective effect on RV function once the patient develops PH.^27^ Analysis of the REVEAL registry found obese PAH patients had a reduced risk of death, and other studies of a number of PH populations have shown obesity confers a survival benefit.^2,28,29^ However, in contrast to this, analysis of PAH patients in Scotland over a 20-year period support a French study suggesting there is no protective effect from obesity in this disease.^1,3^ Furthermore, analysis of PAH patient data from the National Institute of Health-PH registry observed that the best survival was in the overweight patients rather than obese.^29^ These studies challenge whether an obesity paradox exists in PAH.

### Animal models of obesity

A variety of animal models are available to study the effects of obesity on the development and progression of PH. For instance, leptin-deficient *ob/ob* mice lack functional leptin, are grossly overweight and hyperphagic, particularly at young ages, and develop severe insulin resistance.^30^ The *ob/ob* genotype has been shown to result in the spontaneous development of PH in both male and female mice, independent of left-side heart dysfunction and to recapitulate many of the histological features of PH.^7,31^ This is consistent with the observation that in humans, increased BMI is associated with an increase in pulmonary arterial systolic pressure in both males and females.^24^ However, others have observed that *ob/ob* mice are protected against hypoxia-induced PH due to a decrease in proliferation of their pulmonary artery smooth muscle cells (PASMCs).^32^

Similar to *ob/ob* mice, Zucker fatty (ZF) rats have a mutated leptin receptor leading to hyperphagia with obesity apparent from around three weeks of age. ZF rats do not become hyperglycemic but are hyperlipemic, hypercholesterolemic and hyperinsulinemic and develop adipocyte hypertrophy and hyperplasia.^33^ The ZF rat model of obesity also demonstrates hallmarks of PH when aged to five months, with increases in mean pulmonary artery pressure, RV hypertrophy and pulmonary vascular remodelling observed.^34^ The Zucker diabetic fatty (ZDF) rat is a substrain of the ZF rat, which was derived from hyperglycemic ZF rats to gain a model with diabetic features.^35^ ZDF rats also develop a PH phenotype at five months of age.^36^

In addition to genetic models of obesity, high-fat diet (HFD) can also be used to induce obesity in animals. Kelley et al.^37^ demonstrated that 20 weeks of HFD feeding in mice results in the development of a pulmonary hypertensive phenotype with elevated RV pressure, pulmonary vascular resistance, and inflammation. Similarly, others have shown the development of obesity-related PH following HFD feeding in mice with a range of severity in PH phenotype depending on the fat content of the diet and the length of the feeding regime.^38,39^ Pulmonary hypertensive phenotypes can also be observed in certain strains of HFD-fed rats.^34^

It has also been reported that HFD alone does not induce PH per se but can be a modifying factor resulting in an increase in the severity of hypoxia-induced PH and the penetrance of PH in BMPR2 mutant mice.^7,40^ This gives some support to the hypothesis that obesity may act as a ‘second hit’ in some individuals and lead to the development of PAH.

Many researchers use a diet containing 60% fat by calories to create obesity in mice as they become more obese in a shorter period, thus reducing caging costs. In humans, a typical American or European diet will contain ∼36% to 40% fat by energy. Therefore, a tolerable high-fat human diet might contain 50% to 60% of energy as fat.^41^ However, a 60% fat rodent diet presents a much greater distortion of the fat content of a normal rodent chow which normally contains 10% fat. Thus, the use of the use of diets which contain 40% to 45% of fat in rodent studies may be more relevant to human physiology.

A metabolomics study to measure changes in metabolism in mice fed with different HFDs found a range of significantly altered metabolites including free fatty acids, energy metabolites, amino acids and nicotinamide adenine dinucleotide pathway members. A comparison of these effects between liver, kidney and lung found that few changes were shared across organs, suggesting the lung is an independent metabolic organ and that obesity may have direct mechanistic effects on the lung metabolome that can contribute to disease pathogenesis.^42^

It should also be noted that adipose depots in rodents do not perfectly correlate with those in humans. For instance, the omentum contains a large percentage of visceral fat in humans, a depot which is scarcely present in rodents. Conversely, the large epididymal fat pads of male mice are frequently sampled as representative of visceral fat but do not exist in men.^43^

## Mechanisms underlying obesity-associated PAH

Obesity and PAH share several common pathophysiological mechanisms. The initiation of these pathways in obesity may contribute to an environment that predisposes obese individuals to the development of PAH.

### PAH, inflammation and obesity

Inflammation contributes to both an individual’s susceptibility to PAH and to the progression of vascular remodelling in established PAH.^44^ Autoimmune conditions such as systemic lupus erythematosus, systemic sclerosis and other connective tissue diseases are associated with an increased incidence of PAH, and a wide array of inflammatory markers such as IL-1β, IL-2, IL-4, IL-6, IL-8, IL-10, IL-12, IL-18 and TNFα are increased in the serum of PAH patients.^45^ The levels of these inflammatory markers correlate with disease severity or patient survival.^46^ Various studies have also demonstrated perivascular inflammation in PAH lung tissue characterized by the presence of numerous immune cells including macrophages and monocytes, lymphocytes, cytotoxic and helper T cells, dendritic cells and mast cells.^47^ These cells are also present in the occlusive plexiform lesions in the pulmonary vasculature of PAH patients. A direct role for perivascular inflammation in the pathogenesis of PAH has been suggested as pulmonary perivascular inflammation has been shown to correlate with intima and media remodelling.^47,48^

As obesity results in both an enhanced local and systemic inflammatory state, this may provide a link between the conditions and therefore contribute to the development and progression of PAH in obese individuals.

### PAH, oxidative stress and obesity

The pulmonary vasculature undergoes morphological changes in PH that can be mediated by oxidative stress. We and others found the expression of various oxidative stress markers to change in the lungs and pulmonary vasculature in both preclinical models^7,49^ and clinical PAH.^50^ In the lungs, endothelial cells, neutrophils, eosinophils, alveolar macrophages and alveolar epithelial cells are all sites of ROS production facilitating the pulmonary vasculature to generate ROS by complexes in the cell membrane, the cytoplasm, peroxisomes and mitochondria. Enzymes such as nicotinamide adenine dinucleotide phosphate (NADPH) oxidase, uncoupled nitric oxide synthase, dysfunctional mitochondria and xanthine oxidase have all been implicated in generating the increased ROS associated with PAH.^51^

ROS influence the production of a variety of factors implicated in the pathogenesis of PAH and contribute to vasoconstriction and remodelling of the pulmonary circulation. ROS decrease the expression and/or function of redox-sensitive voltage-gated K^+^ channels (Kv1.5, Kv2.1), ultimately leading to Ca^2+^ influx and smooth muscle cell contraction through the activation of myosin light chain kinase and the subsequent phosphorylation of the myosin light chain.^52^ ROS can also mediate vasoconstriction via the activation of the small GTPase, RhoA and its downstream effector, Rho kinase.^53^ Furthermore, ROS upregulate potent pulmonary vasoconstrictors such as endothelin-1 (ET-1) and thromboxane A2 while attenuating the levels of vasodilators, such as prostacyclin and peroxisome proliferator-activated receptor gamma (PPAR-γ).^51^ We have demonstrated that ROS can also contribute to estrogen- and serotonin-induced PASMC proliferation and vascular remodelling. These mechanisms involve NADPH oxidase 1-mediated ROS production and nuclear factor (erythroid-derived 2)-like 2 (Nrf-2) dysregulation that contribute to increased posttranslational oxidative modification of proteins and activation of redox-sensitive signalling pathways.^49,54^

As mentioned previously, increased evidence of ROS production is often observed in obesity, and increased oxidative stress has also been demonstrated in the lungs of mice with obesity-induced PAH.^7^ Thus, oxidative stress and ROS production in obesity, particularly in fat depots in close proximity to the pulmonary circulation, may influence vasoconstriction and the proliferative phenotype of cells of the pulmonary vasculature, initiating or contributing to pulmonary vascular remodelling in PAH.

### PAH, adipokines and obesity

Originally thought to be inert, adipose tissue is now recognized as an important endocrine organ releasing an abundance of bioactive mediators called adipokines. Adipokine production allows communication between adipocytes and other tissues. The plethora of adipokines identified has the capacity to influence every organ system in the body and mediate a diverse array of physiological functions including metabolism, immunity, behaviour, reproduction and cardiovascular function.^19^

Similar to obesity, serum leptin levels have been shown to be elevated in patients with idiopathic PAH (IPAH) and scleroderma-associated PAH independently of proinflammatory cytokines. Leptin may play a role in the immunopathogenesis of IPAH by inhibiting the function of regulatory T cells. Furthermore, leptin is present in lung tissue, and a more intense immunoreactivity for leptin in the endothelium of distal pulmonary arteries has been observed in IPAH patients *versus* controls. Indeed, pulmonary artery endothelial cells contribute to the increased secretion of leptin in IPAH patients.^55^ Increased leptin receptor expression has also been shown in PASMCs from IPAH patients, and these cells are more proliferative to leptin.^56^ In PAH, plasma leptin levels are directly associated with BMI and lower leptin levels, when adjusted by BMI, are associated with an increased overall mortality. The leptin/BMI ratio also acts as a high negative predictive value for mortality at two years. Therefore, leptin levels can predict survival in PAH.^57^

The adipokine, adiponectin, may provide a link between obesity and PAH due to its protective role in the pulmonary circulation. Animal models demonstrate the ability of adiponectin to modulate pulmonary vascular tone, inflammation and remodelling. For instance, adiponectin-deficient mice have reduced levels of endothelial cell nitric oxide in their vascular wall and develop an age-dependent increase in pulmonary artery pressure when compared with wild-type mice.^58^ Another important function of adiponectin is to tonically suppress vascular inflammation. This is exemplified in adiponectin-deficient mice, which develop a spontaneous phenotype characterized by activated lung endothelium, age-dependent increases in perivascular inflammatory cell infiltration and elevated pulmonary artery pressures.^58^ Furthermore, adiponectin can also inhibit vascular smooth muscle cell proliferation by inhibiting growth factor-mediated activation of mammalian target of rapamycin (mTOR) via adenosine monophosphate-activated protein kinase (AMPK) activation.^59^ Similarly, adiponectin-deficient animals develop more prominent pulmonary vascular remodelling in hypoxia-induced PAH.^60^ By direct inhibition of AMPK/mTOR and Nuclear Factor Kappa B (NFκB) pathways, adiponectin also has anti-inflammatory properties and indirectly increases circulating levels of apolipoprotein E (ApoE) and expression of the PPARγ receptor in the lung.^58,61^

Adiponectin is highly abundant in the circulation of lean healthy individuals; however, levels of adiponectin decrease with increasing body mass, and circulating adiponectin levels are decreased in obesity. The impaired production of adiponectin by adipocytes in obesity is the result of oxidative and endoplasmic reticulum stress and the activation of inflammatory cytokines that are prevalent in the adipose tissue of the obese individuals.^62^ Therefore, the reduction in adiponectin in obesity may be influential in the development of PAH by impeding its ability to modulate vascular tone, regulate inflammatory responses and attenuate vascular smooth muscle cell growth

However, adiponectin levels are elevated, rather than decreased, in several chronic inflammatory diseases. The reason for this paradoxical behaviour is unclear. Several studies have also reported an increase in adiponectin levels in PAH patients rather than a reduction confounding its role in PAH.^63,64^ Insulin resistance is prevalent in PAH patients, and elevated levels of insulin have been shown to downregulate AdipoR1/R2 expression limiting adiponectin’s physiological effect and resulting in adiponectin resistance.^65^ Therefore, the increased circulating levels of adiponectin in PAH may be a result of the efforts of adipose tissues to prevent adiponectin functional resistance.

Although circulating levels of adiponectin are increased in PAH, concentrations within the pulmonary circulation are unknown. Interestingly in patients with PH associated with congenital heart disease, although serum levels of adiponectin are increased, adiponectin levels within endothelial cells are decreased,^59^ suggesting the concentration of adiponectin in specific microenvironments may be important in contributing to PAH pathogenesis.

Apelin is an endogenous peptide identified as a ligand of the G protein-coupled receptor APJ and is widely expressed throughout the body but is preferentially produced by visceral adipose tissue with insulin-sensitizing effects.^66,67^ The apelin/APJ system is involved in many physiological processes, such as regulation of blood pressure, cardiac contractility, angiogenesis and energy metabolism. Apelin production is also altered in obesity, with changes in serum apelin detected in multiple tissues in obese patients compared to non-obese controls.^66^ These results suggest that apelin might play an important role in obesity as apelin inhibits lipolysis in adipocytes^68^ and is involved in angiogenesis in adipose tissue.^69^

Apelin is expressed in the lung, localizing in the endothelium of pulmonary arteries, whilst the apelin receptor is present in both endothelial and smooth muscle cells in the vasculature.^70,71^ Apelin is a prosurvival factor for pulmonary artery endothelial cells and can suppress proliferation and induce apoptosis of PASMCs.^72^ Reduced apelin expression has been observed in plasma and pulmonary artery endothelial cells from patients with IPAH and has been attributed to their reduced BMPR2 expression, suggesting that apelin could be effective in treating PAH by rescuing BMPR2 and pulmonary artery endothelial cell dysfunction.^71,72^ A small randomized double-blind placebo-controlled study of acute apelin administration in PAH patients during right heart catheterization has also reported an improvement in cardiac output and pulmonary vascular resistance.^73^ Although changes in apelin production occur in both PAH and obesity, studies investigating apelin expression in obesity are inconsistent with both increases and decreases in plasma apelin reported.^66^ However, as with other mediators, circulating levels are not always indicative of changes in the local microenvironment of the pulmonary circulation and the adipose tissue in its close proximity, and there is still potential for obesity-mediated changes in apelin expression to play a role in the development of obesity-related PAH.

### Obesity, insulin resistance and PAH

Insulin resistance occurs when insulin-sensitive tissues fail to respond to insulin, a phenomenon that is often observed in obesity. Insulin resistance in obesity is manifested by decreased insulin-stimulated glucose transport and metabolism in adipocytes and skeletal muscle and by impaired suppression of hepatic glucose output. As mentioned previously, several mediators contribute to the development of obesity-related insulin resistance, including inflammation, lipotoxicity and hypoxia (Fig. 1).^74^

Insulin resistance has also emerged as a potential mechanism related to the pathogenesis of PAH. Based on retrospective data from the National Health and Nutrition Examination Survey, female participants with a diagnosis of PAH (irrespective of cause) were nearly twice as likely to be insulin resistant (defined as a triglyceride/HDL cholesterol ratio >3.0).^75^ Similarly, an increased incidence of the metabolic syndrome (characterized by insulin resistance, abdominal obesity and hypertension) was observed in patients with pulmonary venous hypertension, a well-defined cause of PH in patients with left heart disease (WHO Class II).^76^ A subsequent study found that 56% of PAH patients had insulin resistance, with 15% of patients having unrecognized type 2 diabetes mellitus (defined as hemoglobin A1c (HbA1c) ≥ 6.0% and  ≥ 6.5%, respectively).^77^ In addition, there was a trend towards lower mean 6-minute walk distance (6MWD) in patients with elevated HbA1c but no significant difference in six-month event-free survival (defined as death, transplantation, hospitalization for right heart failure or acute exacerbation of PAH, or addition of new vasodilator therapy).^77^ Furthermore, increased glucose metabolism has been observed in the lungs of IPAH patients.^78^ However, it has yet to be determined whether the relationship between obesity, insulin resistance and PAH simply represents an association or a cause-and-effect relationship.

Mutations in the BMPR2 gene are a major cause of heritable PAH (HPAH) (present in ∼80% cases) and have also been reported in ∼25% to 30% of IPAH patients.^79^ An association between BMPR2 mutations and insulin resistance has been reported in PAH. A high level of insulin resistance has been observed in mice with an inducible BMPR2 mutation and was associated with rapid weight gain.^80^ In this model, insulin resistance was present prior to the development of PAH. The link between BMPR2 dysfunction and insulin resistance is thought to be mediated by PPARγ, a downstream target of BMPR2.

Genes targeted by PPARγ encode many of the proteins implicated in the pathogenesis of PAH including ET-1, IL-6 and adiponectin.^62,81^ In addition, reduced PPARγ expression has been observed in lungs and circulation of PAH patients.^82^ Thus, there is increasing evidence that gene regulation by PPARγ plays an important role in PAH; indeed, PPARγ agonists have demonstrated therapeutic potential for PAH in preclinical studies.^81^

The BMPR2 dysfunction that occurs during PAH leads to decreased PPARγ activity, increased mitogen-activated protein kinase activity and subsequent stimulation of pulmonary vascular remodelling via the platelet-derived growth factor-β (PDGFR-β) pathway.^40,83^ Given that BMPR2-mediated PPAR-γ activation occurs earlier than Smad1/5/8 phosphorylation, this appears to be independent of the Smad signalling pathway. ApoE and adiponectin are also important downstream effectors of PPARγ, both of which inhibit smooth muscle cell proliferation by converging on the PDGFR-β pathway.^84^ Reduced ApoE expression has been observed in the lungs of IPAH patients, suggesting that the BMPR2/PPARγ/ApoE axis may be a key mediator of the association between insulin resistance and PAH.^85^

PPARγ is abundant in adipose tissue that plays a prominent role in adipogenesis and fatty acid storage and is an active modulator of insulin resistance.^81^ Activation of PPARγ in white adipose tissue enhances its ability to store fatty acids, thus preventing their accumulation in ectopic tissues and reducing the potential for insulin resistance to develop.^86^ Obesity has been reported to inhibit the expression and activity of PPARγ contributing to the development of insulin resistance in obese individuals.^87^ Furthermore, activation of PPARγ in adipose tissue may impact systemic insulin sensitivity by altering the production of adipokines. Transcription of adiponectin is upregulation by PPARγ activation, and studies have shown that plasma adiponectin levels directly correlated with insulin sensitivity.^59^ Activation of PPARγ in adipocytes is also associated with decreased production of TNFα and resistin^88^ which has been shown to improve insulin sensitivity. Furthermore, thiazolidinedione (TZD) antidiabetic drugs act as PPARγ agonists and have been shown to increase the expression of adiponectin and decrease levels of resistin and TNF-α human subjects.^86^ TZDs, pioglitazone and rosiglitazone, have already demonstrated therapeutic potential for PAH in both preclinical and clinical trials.^89–91^ Beyond the TZDs, nitro-oleic acid (NO_2_-OA) and its isomer CXA-10 (10-nitro-9(E)-octadec-9-enoic acid) have demonstrated therapeutic potential through stimulation of PPARγ, upregulation of Nrf-2 and inhibition of NFκβ. CXA-10 has successfully demonstrated safety in preclinical toxicology and Phase I studies, and Phase II studies in PAH are currently underway (NCT04053543, NCT03449524).^92^

Evidence from animal models with BMPR2 mutations suggests insulin resistance develops before PAH and that insulin resistance has a causative role in the development of pulmonary vascular disease.^80^ As insulin resistance is common in obesity, this may provide another mechanism linking obesity to the development of PAH.

As can be seen in Fig. 1, recruitment of inflammatory cells, localized inflammation, alterations in adipokine secretion and insulin resistance are all interlinked in obese individuals and contribute to metabolic syndrome and systemic disease. The activation of these pathways may also contribute to the onset of obesity-related PAH as these pathways are also active in many PAH patients. As discussed later in this article, the changes in adipose tissue and increased adipose deposition in close proximity to the pulmonary circulation are likely to have a profound effect on the local microenvironment in the lung. In particular, the changes in inflammatory mediators and adipokines are known to impact on the expression of the estrogen-synthesizing enzyme aromatase that has been implicated in the pathogenesis of PAH.

## Adipose-derived estrogens and PAH

Estrogens are steroid hormones and play key role in the development of secondary sex characteristics. They are also important in regulating memory and bone density and have been shown to have cardiovascular effects. The three major estrogens are estrone, estradiol and estriol. Premenopause, estrogen synthesis occurs mainly in the ovarian follicles and corpus luteum. In postmenopausal women and in men, estrogen is instead produced by extragonadal sites including adipose tissue where it acts locally in a paracrine fashion or can be released into the circulation. After menopause, adipose tissue is the primary source of estrogen production in the body.^6^

**Fig. 2.**
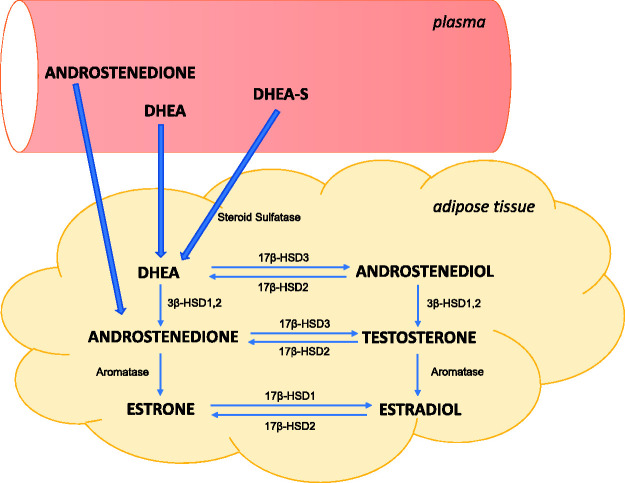
Local production of estrogens in human adipose tissue from circulating precursors. DHEA: dehydroepiandrosterone; DHEA-S: DHEA sulfate; 17β-HSD: 17β-hydroxysteroid dehydrogenase; 3β-HSD: 3β-hydroxysteroid dehydrogenase.

Adipose tissue interconverts stored or circulating sex steroids but does not synthesis sex steroids de novo. Circulating C19 steroid precursors androstenedione, dehydroepiandrosterone (DHEA) and DHEA-sulphate (DHEA-S) act as a reservoir for extragonadal estrogen synthesis.^6^ In particular, aromatase, 17β-hydroxysteroid dehydrogenases (17βHSDs) and CYP1B1 are highly expressed in adipose tissue stromal cells and preadipocytes.^93^ Aromatase (CYP19A1) is a member of the cytochrome P450 superfamily and synthesizes estrogens through the aromatization of androgens, specifically testosterone and androstenedione, resulting in the formation of estradiol and estrone, respectively. 17βHSD mediates the conversion of weak androgens or estrogens to their more potent counterparts: androstenedione to testosterone and estrone to estradiol (Fig. 2).^6^

Given the mass of adipose tissue, the relative contribution of adipose tissue to whole body steroid metabolism is significant. BMI is positively associated with tissue levels of estrogens.^6^ Thus, as fat mass increases in obesity, aromatase expression and, consequently, estrogen levels are also elevated, an effect that is more prominent in postmenopausal women as after menopause adipose tissue is the primary source of estrogen production in the body.^94–96^ Adipose tissue can contribute up to 100% of circulating estrogen in postmenopausal women and 50% of circulating testosterone in premenopausal women. The ratio of 17βHSD to aromatase is positively correlated with central adiposity, implicating increased local androgen production in visceral adipose tissue.^93,97^ Thus, adipose tissue is an important site for both metabolism and secretion of sex steroids.

### Obesity and aromatase

Many obesity-related factors including changes in the profile of inflammatory mediators and adipokines can modulate aromatase gene expression resulting in its upregulation. Expression of aromatase in extragonadal tissues such as adipose is regulated by tissue-specific promoters, so aromatase action can generate high local levels of estrogen with significant biological influence, without significantly affecting circulating levels.^98^ The link between obesity and changes in estrogen synthesis and metabolism have been extensively studied in the context of breast cancer.

**Fig. 3.**
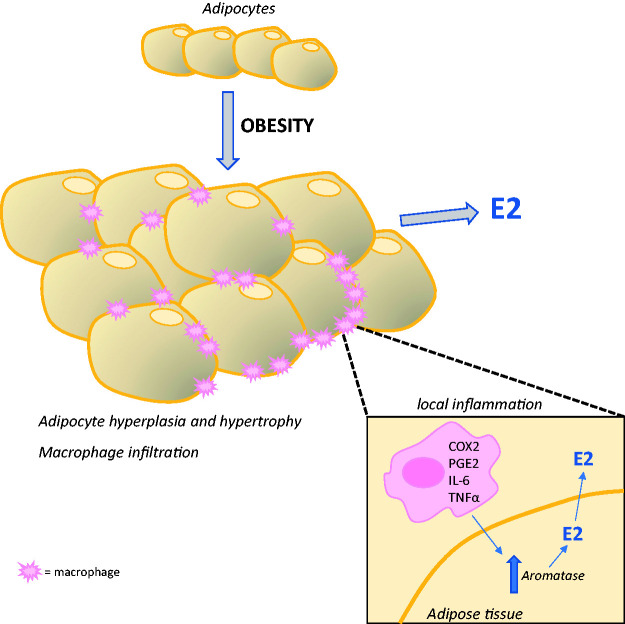
Mechanisms mediating the upregulation of aromatase and estrogen (E2) production in adipose tissue. Increased local inflammation due to macrophage infiltration in obese adipose tissue results in the production of inflammatory mediators known to induce the transcriptional upregulation of aromatase resulting in increased E2 production. COX2: cyclooxygenase-2; E2: estrogen; IL-6: interleukin-6; PGE2: prostaglandin E2; TNFα: tumour necrosis factor-α.

A number of studies have investigated the association between obesity and local estrogen production, identifying several factors dysregulated in obese adipose tissue that induce aromatase expression in adipose stromal cells. A variety of proinflammatory mediators and cytokines (e.g. prostaglandin E2 (PGE2), TNFα, IL-1, IL-6 and cyclooxygenase-2) that are elevated in obesity are known to regulate estrogen production in adipose tissue by upregulating aromatase expression (Fig. 3).^6^ For instance, elevated PGE2 levels in obesity may inhibit p53 which is a negative regulator of aromatase expression resulting in elevation in aromatase.^99^ Furthermore, IL-6 in serum of obese individuals was found to induce PGE2 secretion from breast cancer cells, which in turn induced aromatase expression in primary adipose stromal cells.^100^ PGE2 can also increase aromatase expression by inhibiting the LKB1/AMPK pathway, removing its inhibitory effects on cAMP-responsive element-binding protein-regulated transcriptional coactivators, thus resulting in the upregulation of aromatase.^101^

Adipokines also play a role in regulating aromatase expression. The LKB1/AMPK pathway is normally activated by adiponectin, leading to suppression of aromatase transcription.^102^ However, adiponectin secretion is markedly reduced in obesity and may therefore result in an increase in aromatase expression. Furthermore, leptin levels are increased in obesity and result in the inhibition of p53 and the subsequent upregulation of aromatase.^103^

Therefore, aromatase and estrogen production are increased in dysfunctional obese adipose tissue, at least in the context of breast cancer. However, BMI does not account for volume or type of body fat and may therefore not be the best predictor of local estrogen levels in obesity. This is highlighted by a study showing that inflammation and other systemic markers of metabolic syndrome in white adipose tissue from the breast of women with a normal BMI strongly correlate with aromatase expression and activity.^104^

### Obesity, estrogens and PAH

Female sex is a clear risk factor for PAH and has given rise to the hypothesis that female sex hormones, primarily estrogens, may play a causative role in the development of the condition.^105^ Single nucleotide polymorphisms in the genes encoding aromatase and ESR1, which result in elevated estrogen production, have been associated with an increased risk of portopulmonary hypertension (PPHTN).^106,107^ Expression of aromatase has also been identified in lung tissue and pulmonary arteries of animal models and in patients with PAH, localizing mainly to the vascular smooth muscle.^108^ The concentration of estrogen in the pulmonary artery may therefore be much greater than circulating concentrations due to local synthesis and exert a powerful influence on the pulmonary vasculature. In support of this, levels of estrogen in human PASMCs derived from PAH patients are high.^108^ Interestingly, females were found to express significantly higher levels of aromatase in lung tissue and in PASMCs than males. Indeed, the increased capability of female PASMCs to produce estrogen locally via aromatase contributes to a reduction in the BMPR2 signalling axis and may contribute to the pathology and increased incidence of the disease in females.^108,109^

Sexual dimorphism in the role of aromatase and estrogen can also be seen in animal models of PH. Inhibition of endogenous estrogen synthesis with anastrozole reduces moderate and severe experimental PH and restores BMPR2 signalling in female animals but not male.^108^ Inhibition of endogenous estrogen with anastrozole and fulvestrant also has beneficial effects in female BMPR2 mutant mice,^110^ and metformin can exert therapeutic effects in females SUGEN/hypoxia rats, in part via aromatase inhibition.^111^ Clinically, circulating estrogen levels are elevated in men and postmenopausal women with IPAH.^112,113^

The therapeutic potential of aromatase inhibition has also been demonstrated in a small-scale clinical trial using anastrozole. In this study, anastrozole was found to be safe, well tolerated and improved 6MWD in postmenopausal women and men.^114^ Notably, many of the participants enrolled in this study were overweight or obese.

Endogenous estrogens can also play a role in experimental PH in obese mice. Both male and female leptin-deficient *ob/ob* mice spontaneous develop PH, and we have shown that this can be attenuated by inhibiting endogenous estrogen production using the aromatase inhibitor anastrozole. In this study, aromatase expression in visceral adipose tissue was significantly higher in lean females than males. Furthermore, a marked increase in aromatase expression in visceral adipose tissue was observed in obese males but not female.^7^ We have previously demonstrated that anastrozole treatment is only therapeutic in female hypoxic rodents and not males, suggesting endogenous estrogens play a more prominent role in the development of PH in females. However, we showed that anastrozole can have therapeutic effects in male hypoxic mice that are obese, and this effect is likely due to increased peripheral production of estrogen and its metabolites.^7^ Therefore, in addition to clinical observations, these finding provide further evidence that endogenous estrogens are involved in the development of PAH males, especially in the presence of modifying factors such as obesity.

Obesity may particularly predispose males to the development of PAH due to obesity-mediated adipose dysfunction resulting in altered estrogen production and metabolism.^7^ OSA is common in obese men,^115^ in which elevated circulating estrogen levels occur because of the high expression and activity of aromatase within adipose tissue.^116,117^ OSA is also associated with the development of WHO group 3 PH. Thus, changes in aromatase expression and estrogen production clinically and experimentally suggest that endogenous estrogen may contribute to the pathobiology of PAH in both males and females by sexually dimorphic mechanisms.

The enhanced production of estrogen in adipose tissue during obesity and the release of endocrine factors from adipocytes and stromal cells within fat depots can promote cell growth and have been linked with development of various cancers, including breast cancer.^6^ There is thus a strong link between obesity-driven adipose inflammation and estrogen biosynthesis in proproliferative disease. As these signalling pathways converge in obese PAH patients, they may contribute to the development of obesity-related PAH.

### Obesity and CYP1B1

CYP1B1 is a member of the cytochrome P450 enzyme family 1, subfamily B, polypeptide 1 and is constitutively expressed in various tissues including fat, heart and lung. In addition to the oxidation of xenobiotics, CYP1B1 is involved in the metabolism of many important physiological compounds, including estrogen.^118^ Compounds formed following the metabolism of estrogen by CYP1B1 exert a wide array of physiological effects and have been associated with various cancers. CYP1B1 also plays an important role in adipogenesis and obesity. CYP1B1 is highly expressed in white adipose tissue in humans, and its expression increases upon adipogenic stimulation.^119^ A review of obesity-related genome-wide sequencing studies indicated that CYP1B1 was one of three highest scoring genes associated with obesity.^120^ Animal models further indicate a role for CYP1B1 in obesity. An HFD has been shown to increase CYP1B1 expression in adipose tissue in mice, whilst CYP1B1 deficiency attenuates HFD-induced obesity and improves insulin sensitivity without changing calorific intake, suggesting CYP1B1 may modulate energy metabolism.^118^ Furthermore, inhibition of the aryl hydrocarbon receptor, an upstream activator of CYP1B1 expression, resulted in the downregulation of CYP1B1 and inhibited hypertrophy and hyperplasia in visceral adipose tissue, reversing the effects of HFD.^121^

Changes in CYP1B1-mediated estrogen metabolism in obesity have not been well studied. However, we have shown that CYP1B1 is upregulated in white adipose tissue in obese mice and is involved in mediating the production and release of the mitogenic estrogen metabolite 16OHE1.^7^

### Obesity, estrogen metabolism and PAH

**Fig. 4.**
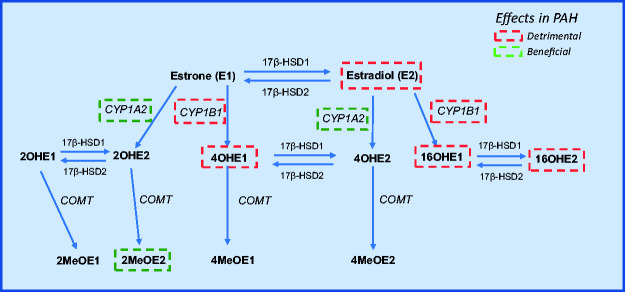
Estrogen metabolism. Estrone (E1) and estradiol (E2) are synthesized by aromatase. Hydroxylation of E1 and E2 occurs at C2, C4 and C16 positions by cytochrome P450 enzymes (the most prominent being CYP1A2 and CYP1B1 promoting beneficial and detrimental hydroxylation, respectively) resulting in the formation of 16α-hydroxyestrogens, 2-hydroxyestogens and 4-hydroxyestrogens by cytochrome P450 enzymes. The 2- and 4- hydroxyestrogens are converted to 2- and 4-methoxyestrogens via COMT. All E1 and E2 metabolites are maintained in equilibrium by 17β-HSD1 and 17β-HSD2 enzymes. PAH: pulmonary arterial hypertension; COMT: catechol-O-methyltransferase; 17β-HSD: 17β-hydroxysteroid dehydrogenase; 16OHE1: 16α-hydroxyestrone; 16OHE2: 16α-hydroxyestradiol; 2OHE1: 2-hydroxyestrone; 2OHE2: 2-hydroxyestradiol; 4OHE1: 4-hydroxyestrone; 4OHE2: 4-hydroxyestradiol; 2MeOE1: 2-methoxyestrone; 2MeOE2: 2-methoxyestradiol; 4MeOE1: 4-methoxyestrone; 4MeOE2: 4-methoxyestradiol.

Estrogen metabolism also plays a key role in PAH. As described above, there is an abundance of CYP1B1 in adipose tissue. CYP1B1 mediates C-16 hydroxylation of estrogen resulting in the formation of the metabolites 16OHE1 and 16α-hydroxyestradiol (16OHE2) (Fig. 4). CYP1B1 over-expression has been observed in PASMCs from both idiopathic and hereditary PAH patients, and various SNPs in CYP1B1 have been associated with increased disease penetrance.^8,122^ Conversely, Epstein-Barr virus (EBV)-immortalized B cells cultured from female HPAH patients have 10-fold lower expression of CYP1B1 than control groups. A reduction in CYP1B1 was not observed in these cells when cultured from male HPAH patient.^123^ This may reflect phenotypic difference in cell type compared to primary cultures of human PASMCs. In addition, estrogen influences B cell maturation and selection and may account for the differences observed in, EBV-immortalized cells and in males and females.^124^ Furthermore, the BMPR2 ligands, BMP2 and BMP4, also have roles in the development, growth potential and apoptosis of B cells.^125^ As the B cells studied were from HPAH patients with dysfunctional BMPR2 signalling, they may be phenotypically altered, resulting in changes to estrogen metabolism and differential expression of CYP1B1 compared to human PASMCs from HPAH patients.

We have shown that serum 16OHE1 and 16OHE2 accumulate in IPAH patients, with 16OHE1 levels relating to disease severity.^126^ The levels of 16OHE1 in PAH patients are sufficient to cause proliferation of human PASMCs.^8,54^ 16OHE1 itself can induce PH in mice.^8^ Pharmacological inhibition of CYP1B1 using tetramethoxystilbene (TMS) also demonstrates therapeutic effects in animal models of PAH^7,8^ and attenuates estrogen-induced proliferation in PASMCs.^8^ In particular, TMS has beneficial effects in a model of obesity-related PAH.^7^ The CYP1B1 metabolite, 16OHE1, also has potent mitogenic effects in the pulmonary circulation *via* mechanisms involving ROS production.^8,54^ 16OHE2 can also cause proliferation of human PASMCs and migration of blood outgrowth endothelial cells derived from PAH patients at concentrations observed in IPAH patient serum.^126^ Additionally, plasma levels of the estrogen metabolite 16OHE2 have been demonstrated to accumulate in patients with PPHTN.^107^

As discussed previously, elevated CYP1B1 is associated with obesity and metabolic syndrome, playing an important role in increasing adiposity and insulin resistance. Furthermore, leptin is known to upregulate CYP1B1 expression in breast cancer cells.^127^ Therefore, the increase in leptin levels in obesity may contribute to the increase in CYP1B1 expression observed. We have shown that CYP1B1 is also highly expressed in adipose tissue, and thoracic adipose tissue from obese mice produces 16OHE1.^7^ This may contribute to the pulmonary hypertensive phenotype of obese mice as TMS can prevent the development of PH in these animals.^7^ The close proximity of thoracic fat to the right ventricle and pulmonary circulation may facilitate interactions with adipose-derived estrogens and create a microenvironment that leads to the development of PAH and/or mediates PAH disease progression.

The estrogen metabolite 2-methoxyestradiol (2MeOE2) is synthesized by catechol-O-methyltransferase (COMT) (Fig. 4). We demonstrated that 2MeOE2 can have beneficial antiproliferative effects in PAH via inhibition of HIF1α and microtubular disruption.^128^ Furthermore, 2MeOE2 shares a structural similarity with PPARγ ligands and thus acts as a PPARγ agonist, stimulates AMPK signalling and increases insulin sensitivity.^129^ COMT deficiency was found to exacerbate the effects of HFD-induced insulin resistance in mice.^130^ Reduced COMT activity and 2MeOE2 levels have been linked to development of obesity and insulin resistance, in addition to PAH.^130,131^ The adipokine leptin has been shown to decrease COMT expression in breast cancer cells.^127^ Therefore, the increased leptin levels observed in obesity may result in a reduction in COMT and a decrease the levels of the 2MeOE2, thus attenuating its protective effects on the pulmonary circulation and resulting in a more proproliferative environment that contributes to the development of PAH.

On the other hand, the proproliferative estrogen metabolite 16OHE1 induces pulmonary vascular remodelling and may promote insulin resistance in PAH.^7,9^ For example, Fessel et al.^9^ found that treating BMPR2-mutant vascular smooth muscle cells with 16OHE1 significantly decreased mobilization of the glucose transporter Glut4 in response to insulin and expression of PPAR-γ and lipid transporter CD36. Based on a proof-of-concept study, 16OHE2 has recently been hypothesized to be a mediator of PAH.^126^ However, much research is required to test this hypothesis *in vitro* and *in vivo*, and whether this has a similar effect to 16OHE1 in promoting insulin resistance is yet to be investigated.

**Fig. 5.**
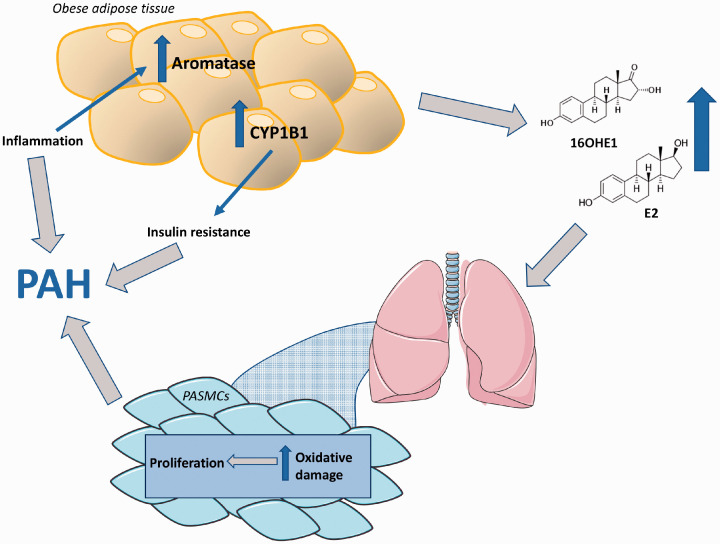
Changes in estrogen metabolism in adipose tissue and its contribution to the development of obesity-related PAH. Obesity causes an increase in the expression of aromatase and CYP1B1 in adipose tissue resulting in the increased production of estrogen (E2) and its metabolite 16OHE1. In particular, 16OHE1 induces proliferation of PASMCs and contributes to pulmonary vascular remodelling in PAH. CYP1B1 is also associated with insulin resistance, another underlying pathology linked with PAH. PAH: pulmonary arterial hypertension; 16OHE1: 16α-hydroxyestrone; PASMCs: pulmonary artery smooth muscle cells.

Evidence is growing for the role of estrogen and its metabolites in the pathogenesis of PAH. Adipose-derived estrogens play a major role in the development of breast cancer, and it is therefore plausible that they also contribute to the proproliferative changes that occur in the pulmonary circulation in PAH. In obesity, the increased production of estrogen by adipose tissue influences circulating estrogen levels that in turn may have direct effects on the lung. Furthermore, changes in adipokines and inflammatory cytokines may also affect the expression of aromatase within lung tissue resulting in changes in the estrogenic profile of the lung and contributing to a proproliferative environment. The upregulation of CYP1B1 in obesity is likely to contribute to an increase in the presence of 16α-hydroxyestrogens that have also been associated with PAH. Elevated CYP1B in obesity contributes to insulin resistance which has also been linked to the development of PAH. Thus, obesity-related changes in estrogen metabolism may contribute directly and indirectly to the pathogenesis of PAH (Fig. 5).

## Contribution of specific fat depots to PAH

The distribution of adipose tissue is of great importance with regards to obesity-related comorbidities. Adipose tissue develops in multiple discrete locations, and the most common classification groups white adipose tissue into subcutaneous and visceral categories. Subcutaneous fat is located in upper and lower body regions and is the most prominent white adipose tissue depot in lean healthy individuals comprising ∼80% of all adipose tissue.^132^ Localized within the visceral compartment, visceral adipose tissue is highly metabolically active and continually releases free fatty acids into the portal circulation.^133^ Many obese individuals accumulate fat intra-abdominally in visceral deposits. This is known as central obesity, and the excess visceral adiposity leads to an array of cardiovascular disease risk factors known as metabolic syndrome.^134^

Evidence from animal models and cultured adipocytes suggests that the preserved expansion capability of subcutaneous white adipose tissue mitigates extensive visceral and hepatic fat accumulation and gives some protection against metabolic disease.^135^ Indeed, lower body subcutaneous white adipose tissue does not correlate with risk factors for metabolic syndrome, potentially due to slower free fatty acid turnover, higher levels of adipocyte hyperplasia and lower levels of inflammation.^136^

In addition to major white adipose tissue depots, distinct tissue-associated depots are distributed throughout the body, including adipocytes within the dermis, skeletal muscle and the epicardial fat pad. These depots are often small, intricate and closely associated with anatomical structures. They perform novel tissue- and organ-specific functions and allow adipocytes to exert profound influence on the neighbouring tissue.^137,138^ Significant regional differences in adipocyte behaviour have been characterized, and recognition of their importance is rapidly growing.^139^ However, how the local microenvironment influences the function of adipose tissue and its impact on systemic metabolism remains largely unexplored.

Growing evidence implicates aberrant inflammation, ROS and estrogen metabolism in PAH pathogenesis, playing an active role in PAH pulmonary vascular remodelling. However, mechanisms that trigger crosstalk between these pathways and components of the pulmonary circulation are still unclear. Could tissue-specific fat depots in close association with pulmonary and cardiac tissue be the link and contribute to the development of obesity-related PAH?

### Perivascular adipose tissue and PAH

Perivascular adipose tissue (PVAT) plays an important role in modulation of vascular physiology. The phenotype of PVAT is distinct from other fat deposits and varies depending on location. Around small vessels PVAT is comprised of adipocytes that are less differentiated and vascularized than typical white adipose tissue. Perivascular adipocytes also express higher levels of angiogenic factors such as vascular endothelial growth factor, hepatocyte growth factor and thrombospondin. In healthy individuals, PVAT normally has protective antiproliferative, anti-inflammatory and anticontractile effects on the vasculature.^140^

The adipocyte dysfunction that occurs in obesity causes inflammation, oxidative stress and hypoxia resulting in a loss of the protective effects of PVAT. For instance, increases in TNFα and ET-1 have been observed in the PVAT of small arteries isolated from biopsies of visceral fat in obese individuals resulting in impaired nitric oxide release.^141^ Dysfunctional PVAT in obese individuals may also result in oxidative stress and the recruitment of immune and inflammatory cells to the perivascular layer of pulmonary arteries contributing to vascular remodelling and endothelial dysfunction in PAH.^142^ Additionally, perivascular adipocyte expansion has also been linked to the development of vascular insulin resistance as it releases a variety of factors including IL-6, IL-8 and Monocyte Chemoattractant Protein-1 (MCP-1) that have been shown to affect insulin sensitivity locally in a variety of vascular beds.^143^ Given the important contribution of insulin resistance to the pathogenesis of PAH, the potential for PVAT expansion in obesity to influence insulin sensitivity may contribute to the development of PAH in some obese individuals. PVAT expansion in obesity within the human lung has not been characterized. However, in the SUGEN/hypoxia rat model of PAH, intense lipid staining in close proximity to the lung vasculature was observed in lung sections of both control and PAH animals. The pattern of staining was localized around the lung vasculature and asymmetric, which suggests the existence of lipid-laden cells within the lung and shows their irregular accumulation in proximity to the lung vasculature.^144^ Further studies in this area are required to elucidate the potential role of PVAT in the pathobiology of PAH.

### Cardiac fat and PAH

Cardiovascular adipose tissue may be of particular importance in the context of PAH. In the thorax, two main adipose depots surround the heart: epicardial and pericardial adipose tissue. Epicardial adipose is particularly important in normal heart function and can contribute to the pathogenesis of cardiovascular diseases. Epicardial fat represents a small fraction of overall visceral fat but can exert a profound effect on cardiac function.^145^

Located between the myocardium and the visceral layer of the pericardium, epicardial fat covers approximately 80% of heart’s surface area. Epicardial adipocytes are similar to white adipocytes in other white adipose tissue depots but express high levels of the Uncoupling Protein 1 (UCP-1) and as a result can actively generate heat. This may provide the heart with thermal protection, in addition to mechanical cushioning.^146^ Furthermore, high levels of free fatty acids are produced by epicardial fat that can directly diffuse to the adjacent myocardium, acting as an additional local energy source.^147^ Epicardial adipose tissue also secretes a plethora of adipokines that have a major effect on the function of the heart and coronary arteries.^145^

Obesity-related dysfunction of the epicardial fat pad can have a profound effect on cardiovascular function. The increase in epicardial adipose tissue deposition and infiltration into the myocardium in obese individuals has been shown to have adverse effects on heart function. Increased mass due epicardial adipose tissue expansion increases the workload on the heart and contributes to cardiac hypertrophy. Additionally, obesity results in an increase in adipose-derived proinflammatory signalling that can have further detrimental effects on heart and vascular function.^147^

Increased lipid deposition has been observed in cardiomyocytes in the right ventricle of pulmonary hypertensive BMPR2 mutant mice. Similarly, lipid deposition has also been found in the failing right ventricles of HPAH patients, suggesting a lipotoxic cardiomyopathy may occur within the right ventricle in PAH.^148^

Therefore, in obese individuals, epicardial adipose tissue and ectopic lipid disposition in cardiomyocytes may contribute to the development of an inflammatory and insulin-resistant microenvironment in close proximity to the right ventricle and pulmonary circulation that has powerful physiological effects resulting in the development and progression of PAH and contributing to a dysfunctional right ventricle.

Thoracic adipose tissue is of particular interest in PAH, as its lymphatics drain directly into the pulmonary circulation and it can exert local and systemic effects.^43^ Interestingly, a recent study investigating the association of thoracic visceral fat with PH in patients with advanced lung disease referred for lung transplantation found that lower levels of thoracic fat were associated with a higher risk of PH.^149^ While obesity is associated with PAH, some local fat depots may produce mediators such as vaspin and adiponectin that have a cardioprotective effects and contribute to obesity paradox observed in some PAH cohorts. Estrogen produced by thoracic fat may also have protective effects on heart function in the context of PAH as animal models of the condition have demonstrated cardioprotective effects of exogenously administered estrogen.^150,151^ For instance, estrogen *via* estrogen receptor α, increases BMPR2 and apelin in the failing right ventricle of experimental PAH.^152^ Human atrial and epicardial adipose tissue expresses aromatase, and in rodents, aromatase-mediated estrogen production is significantly elevated with obesity-related cardiac adiposity and associated atrial arrhythmogenicity.^153^ We have recently demonstrated that thoracic fat from obese mice releases 16OHE1, which is known to play a role in pulmonary vascular remodelling in PAH.^7^ However, further studies are needed to determine the exact contribution of estrogens derived from thoracic fat to right heart function and how they may be involved in PAH.

### Sexual dimorphism in adipose tissue distribution and function

Sexual dimorphism in the distribution of adipose tissue is well documented. On average, women have a higher percentage of body fat than men and store more fat in subcutaneous areas, especially in the gluteal and femoral depots.^154^ Conversely, men accumulate fat preferentially in upper‐body and visceral compartments.^155^ At comparable levels of total adiposity, women have more subcutaneous adipose tissue both in the abdominal and in the gluteofemoral area.^5^ Furthermore, functionally active areas of brown adipose tissue are present more frequently in women than in men.^156^ The preferential distribution on fat in subcutaneous gluteal and femoral fat depots in women is also associated with lower metabolic risk.^154^

Evidence suggests that sex steroids play an essential role in fat distribution as sex differences in adiposity emerge during puberty and menarche and tend to diminish at menopause, when female sex hormone patterns change and fat distribution in women shifts toward that of men, and women develop more central obesity that contributes to an increase in their incidence of cardiovascular and metabolic disease.^157^ Evidence suggests that estrogen, acting directly or through its receptors, can differentially augment sympathetic tone resulting in lipid accumulation in the subcutaneous depot in women and the visceral compartment in men. Estrogens also influence the expandability of adipocytes enhancing the expandability of subcutaneous adipose tissue whilst inhibiting the expansion of visceral depots.^158^ Therefore, the reduction in estrogen levels likely contribute to the changes in adipose tissue distribution following menopause.

An increase in the deposition of fat around the heart and aorta has been observed in peri- and postmenopausal women independent of age, obesity and other covariates and is associated significantly with a decline in circulating estrogen levels.^159^ Furthermore, data from the Framingham Heart Study found associations between pericardial and periaortic fat and coronary heart disease risk factors are significantly stronger in women than men,^160,161^ suggesting these fat depots play a role in the higher risk of coronary heart disease reported in women after menopause. Major PAH registries have recently reported an increased mean age of PAH onset, with the US REVEAL and European Comparative, Prospective Registry of Newly Initiated Therapies for Pulmonary Hypertension (COMPERA) registries reporting the mean age of onset as 53 and 59 years, respectively; therefore, many women will be peri- or postmenopausal at time of diagnosis.^162,163^ No information on changes in adipose tissue distribution during menopause have been documented in PAH cohorts, but given that the increase in visceral adipose deposition that occurs in women during this time is associated with the occurrence of other cardiovascular diseases, it is likely it may also be associated with PAH.

Numerous studies have established that there are sex-dependent differences in the function of adipose tissue. For instance, women have higher circulating adiponectin levels compared with men.^164^ Animal models have also demonstrated sexual dimorphism in the function of adipose depots and adipokines within the cardiovascular system. Elevated levels of resistin in PVAT from males compared to females contribute to the sex-dependent differences in resistance vessel function in the Spontaneously Hypertensive Stroke-Prone Rat resulting in a reduction in the protective effect of PVAT.^165^ Estrogen has been shown to downregulate resistin expression both *in vivo* and *in vitro* and may account in part for the sex-differences observed in PVAT function.^166^ Additionally, PVAT surrounding porcine coronary arteries from females has an anticontractile effect mediated by adiponectin that is not observed in males, proving further evidence that PVAT may function differently in males and females.^167^

Currently, few studies have investigated the role of specific fat deposits and the mechanisms by which they may contribute to obesity-related PAH. Adipose tissue is a major source of estrogen production, and sexual dimorphism has already been reported in signalling mediated by estrogens in the PAH pathogenesis. As discussed above, estrogen is also thought to mediate many of the sex differences in adipose tissue distribution. Studies phenotyping adipose distribution in male and female PAH patients may yield some clues as to the contribution of specific fat depots to PAH. Given the large number of women diagnosed with PAH, the contribution of changes in adipose tissue distribution and function observed during menopause is also highly relevant. A full understanding of the mechanisms by which adipose tissues accumulate in specific depots and how they differ metabolically depending on sex may give important insight into how obesity-related PAH develops and yield novel therapeutic strategies.

## Adipose-derived estrogens as a therapeutic target

Given the growing levels of obesity and the pivotal role of adipose tissue in metabolic health and disease, adipose tissue has potential as a direct or indirect therapeutic target in the treatment of obesity-related PAH. The substantial role of adipose tissue in estrogen production suggests the modulation of sex steroids may be of particular benefit in the treatment of PAH. In particular, obese PAH patients may respond well to any novel therapies that reduce the influence of estrogens. Drugs that inhibit estrogen synthesis and attenuate the effects of estrogens are now in clinical trials for PAH.

Aromatase inhibitors are already widely used in the treatment of estrogen-sensitive breast cancer. Genetic variations in estrogen signalling have been associated with PAH.^106^ In addition, men and postmenopausal women with PAH have higher levels of estrogen compared with controls, and this correlates with shorter 6MWD.^168,169^ These data have led to clinical trials of treatments targeting the sex hormone profile of PAH patients. A small placebo-controlled randomized clinical trial of the aromatase inhibitor anastrozole showed a reduction in circulating estrogen levels and a significant increase in 6MWD over a 12-week period.^114^ A larger Phase II randomized clinical trial of anastrozole in PAH is currently enrolling (NCT03229499).^170^

Increased expression of estrogen receptors has been observed in PASMCs from PAH patients, with estrogen receptor alpha (ERα) increased in females, whilst estrogen receptor beta (ERβ) is increased in males.^171^ This makes blocking estrogen receptors an attractive target to mitigate the effects of estrogen in the pathogenesis of PAH. Fulvestrant is an estrogen receptor antagonist currently used in the treatment of breast cancer that can inhibit the function and decrease the expression of estrogen receptors. In a small proof-of-concept study (NCT02911844), fulvestrant administration resulted in a higher 6MWD, increased stroke volume and a decrease in 16OHE2 levels in postmenopausal women with PAH.^172^ The reduction in 16OHE2 may account for some of the beneficial effects observed as this estrogen metabolite has previously been linked to PPHTN.^107^ Combined administration of fulvestrant and anastrozole has also been shown to result in a marked improvement in hemodynamics and pulmonary vascular remodelling in a BMPR2 transgenic mouse model of PAH.^110^

Tamoxifen is the most commonly used selective estrogen receptor modulator (SERM) and acts as an antagonist at ERα and ERβ, although it can have a partial agonist effect in some tissues. It has been shown to have therapeutic effects in a murine model of PAH.^110^ A small, double-blind, randomized, placebo-controlled, proof-of-concept Phase II trial to determine the safety of tamoxifen in males and pre-and postmenopausal females is currently underway (NCT03528902). Due to their strong antiestrogenic nature, fulvestrant and anastrozole are regularly used in postmenopausal women and not recommended for use in premenopausal women due to induction of menopause. As tamoxifen has some efficacy in preclinical studies and is already widely used in premenopausal women, it is important to determine whether it can be of therapeutic benefit in younger female PAH patients.

CYP1B1 modulators could also be considered as therapeutic agents to protect against the metabolic effects of obesity. TMS is a selective, potent CYP1B1 inhibitor, and its beneficial effects on several metabolic diseases, including tumorigenesis, hypertension, atherosclerosis and adipogenesis, have been determined in animal models.^118^ As discussed earlier, TMS has also shown to have therapeutic effects in animal model of PH, including obesity-induced PH.^7,8^ CYP1B1 inhibition has also been explored clinically as a therapeutic target. ZYC300 is a CYP1B1-based vaccine which stimulates the immune system to elicit a cytotoxic T lymphocyte response against tumour cells expressing CYP1B1. Phase I trials of ZYC300 conducted in late-stage cancer patients have shown some promise.^173,174^

With growing levels of obesity, adipose-derived estrogens and their metabolites are likely to become the major source of estrogens contributing to the development of PAH. Third-generation aromatase inhibitors such as anastrozole have proven more beneficial in suppressing estrogen production in adipose tissue in men than other sources,^175^ suggesting aromatase inhibition may have additional benefits when administered to obese PAH patients. Likewise, given the abundance of CYP1B1 in adipose tissue, its inhibition may confer a greater benefit when taken by obese individuals with PAH.

## Summary

The obesity pandemic has highlighted the importance of adipose tissue, and it is no longer merely considered as a storage organ but is recognized as an important endocrine organ essential in regulating metabolic function. Obesity causes many changes in adipose tissue including adipocyte hypertrophy, infiltration of inflammatory cells, fibrosis and altered adipokine secretion. The resultant adipose tissue dysfunction has a variety of systemic and local effects that are also known to play a role in PAH. Therefore, obesity may create a pathophysiological environment that facilitates disease development in susceptible individuals, and indeed, obesity-related PAH accounts for between 30% and 40% of PAH patients.

Obesity-mediated changes in adipokine and inflammatory cytokines can result in insulin resistance, which is already common in PAH patients. These mediators also affect aromatase expression and estrogen metabolism through CYP1B1, processes that are important in PAH pathophysiology. Ectopic fat deposition in obesity, particularly in peri-vascular and cardiac tissue, provides a direct link between adipose tissue dysfunction and the pulmonary circulation, and more studies investigating the role of these fat deposits in the pathology of PAH are warranted. Adipose tissue distribution and function shows marked sexual dimorphism, and given the large number of women diagnosed with PAH, studies investigating the phenotype of adipose tissue distribution in both sexes are needed.

Increased adiposity and obesity are associated with aging, and the resultant chronic low-level inflammation often causes insulin resistance.^176^ Major PAH registries have recently reported an increased mean age of PAH onset, with the US REVEAL and European COMPERA registries reporting the mean age of onset as 53 and 59 years, respectively.^162,163^ Thus, with a globally expanding older population (with higher rates of insulin resistance, obesity and other comorbidities), the underlying chronic inflammation and adipose dysfunction is likely to play an increasing role in the pathogenesis of PAH.

Whilst current therapeutic strategies for PAH improve exercise capacity, quality of life and long-term outcomes, they are still palliative, and PAH carries a high risk of mortality due to right heart failure. The five-year survival rate for patients suffering from PAH is around 60%.^162^ Thus, new treatments are required, and in the era of personalized and precision medicine, increasing our knowledge of adipose tissue biology coupled with improved phenotyping of adipose tissue distribution and how this contributes to the development of PAH may yield novel therapeutic strategies.

Higher estrogen levels are observed in both sexes and associated with worse functional markers of PAH. This coupled with altered estrogen metabolite profiles where elevated 16α-hydroxyestrogens are likely to contribute to PAH make widely used antiestrogenic drugs with well-established safety profiles an attractive therapeutic strategy. Given the large number of obese PAH patients, the contribution of adipose tissue to this skewed estrogenic profile requires greater investigation, and obesity should be a consideration when tailoring treatments to patients in order to maximize therapeutic benefit.
